# An In Vivo Investigation of Non-Metallic vs. Metallic Hand Scalers on Zirconia Implant-Supported Crowns: A Year-Long Analysis of Peri-Implant Maintenance

**DOI:** 10.3390/jfb15010009

**Published:** 2023-12-23

**Authors:** Dayna L. Roemermann, Reem Atout, Igor Pesun, Anastasia Kelekis-Cholakis, Chrysi Stavropoulou, Stefan N. Renvert, Rodrigo França

**Affiliations:** 1 Department of Dental Diagnostic and Surgical Sciences, Rady Faculty of Health Sciences, University of Manitoba, Winnipeg, MB R3T 2N2, Canada; droemermann@gmail.com (D.L.R.); reem.atout@gmail.com (R.A.); anastasia.cholakis@umanitoba.ca (A.K.-C.); chrysi.stavropoulou@umanitoba.ca (C.S.); 2 Department of Restorative Dentistry, Rady Faculty of Health Sciences, University of Manitoba, Winnipeg, MB R3T 2N2, Canada; igor.pesun@umanitoba.ca; 3 Oral Health Sciences, Kristianstad University, 291 88 Kristianstad, Sweden; stefan.renvert@hkr.se

**Keywords:** dental implants, zirconia, peri-implant maintenance, surface roughness, cytokines

## Abstract

This study examined whether the degree of abutment surface modification that may occur with regular periodontal instrumentation has a clinical impact in terms of increased plaque accumulation and increased peri-implant tissue inflammation on zirconia implant abutments. Thirteen patients who had zirconia implant crowns were recruited in this randomized clinical trial. Each patient acted as their control and had either the buccal or lingual surface of their screw-retained implant restoration scaled with a metallic scaler and the other surface with a non-metallic scaler at 3, 6, 9, and 12 months. Cytokine testing of the peri-implant crevicular fluid was completed at 0, 3, and 12 months for IL-2, IL-4, IL-6, IL-8, IL-10, TNF-α, or IFNγ. Implant crowns were removed at 12 months and evaluated under an atomic force microscope for the average roughness (Ra). The implant crowns were polished and re-inserted. The results were analyzed using the Kruskal–Wallis test, and post hoc tests were conducted with a significance level of α = 0.05. Significant differences in surface roughness (Ra) were observed between the metallic and non-metallic scalers. The median Ra values were 274.0 nm for metallic scalers and 147.1 nm for non-metallic scalers. However, there were no significant differences between the type of scaler used and the amount of clinical inflammation or cytokine production. Metallic scalers produced deeper, more aggressive surface alterations to the abutment/crown zirconia surface, but there was no statistically significant difference between the degree of surface alterations, amount of BOP, and the amplitude of cytokine inflammation produced.

## 1. Introduction

The increasing success rates of dental implants [1] have resulted in an expanding percentage of the population deciding to undergo dental implant treatment to replace missing teeth. Dental implants are a good long-term option for both fully and partially edentulous patients [2], and the success rate has been reported to be 97.5% over 5 years [3]. The projected proportion of patients with a dental implant in 2026 could be as high as 23% [4]. Biofilm accumulation around dental implants can cause peri-implant tissue inflammation, a condition known as peri-implant mucositis [5]. Clinical signs include bleeding on gentle probing, erythema, and swelling or suppuration. There may be an increase in probing depth due to a decrease in resistance to probing of the peri-implant tissues [6]. In some cases, peri-implant mucositis may progress to a more severe inflammatory condition, which involves the loss of bone around the implant fixture, known as peri-implantitis [7]. Osteo-immunoinflammatory mediators are produced by the host response and play a crucial role in the breakdown of peri-implant tissue. These mediators include cytokines, chemokines, growth factors, and enzymes, among others. They are involved in the regulation of bone metabolism, immune response, and inflammation, and their dysregulation can lead to the development of peri-implant diseases such as peri-implant mucositis and peri-implantitis. MMP-8, or collagenase-2, is one of the major mediators of peri-implant tissue destruction and the most prevalent collagenolytic protease in these diseased tissues. It is produced by various cells, including neutrophils, macrophages, and fibroblasts, and is involved in the degradation of extracellular matrix components such as collagen, which is a major component of the peri-implant soft and hard tissues. The presence of MMP-8 in the peri-implant sulcus fluid has been associated with the development of peri-implantitis in response to plaque deposition, and its concentration has been proposed as an early sign of peri-implant breakdown [8]. Good patient oral hygiene practices and a professional peri-implant maintenance program are very important tools in preventing the onset of peri-implant diseases [7].

Titanium has been extensively used for the fabrication of implant abutments due to its strength, resistance to malformation, and the opportunity to fabricate as one piece. However, titanium abutments have been shown to produce a grayish appearance in the overlying tissue. Zirconia abutments offer a more esthetic alternative, especially in esthetic cases or in patients with a thin biotype [9]. In addition, in vivo, zirconia appears to be less prone to bacterial adhesion as compared to titanium [10]. Titanium is a metal and can often suffer corrosion, whereas zirconia is made of ceramic and will not corrode. This could account for healthier gingival tissues around an implant [9].

Instruments used for the debridement of dental implants should be efficient, effective in removing plaque and calculus, and should refrain from causing damage to the abutment/crown surface [11]. During the cleaning of the abutment/crown surface, contact of the implant scaler with the metal surface could create a roughened surface, which may, in turn, increase the propensity of bacterial accumulation and lead to pro-inflammatory changes in the peri-implant tissues [12]. Previous in vitro studies have examined the degree of roughness caused to titanium surfaces following instrumentation with various scalers and ultrasonic tips. Conventional stainless steel scalers have been shown to produce a significantly higher surface roughness when compared to non-metallic and novel metallic implant scalers. Scanning electron microscope images have shown remarkable scratches on the titanium implant surface from conventional stainless steel scalers [11,12,13,14,15,16,17,18,19,20,21,22]. As a result of this, specific implant instrumentation was developed to eliminate surface alterations from occurring. However, the application of non-metallic scalers to the implant surface has been labeled as inadequate in removing bacteria from a roughened implant surface [16]. Metallic scalers have been shown to debride the implant surface more effectively and efficiently [17]. It is important to note that previous studies have been carried out in standardized, in vitro titanium discs or abutments, and the results may not apply to clinical situations [11,12,14,15,16,17,18,19,20,21,22].

Zirconia ceramics was introduced as a dental restorative material due to its excellent mechanical properties [23]. This oxide ceramic possesses a high flexural strength (~1000 MPa), a small surface roughness (~15 nm), and satisfactory chemical resistance that results in good biocompatibility [24,25]. It would be important to investigate whether the degree of abutment surface modification that may occur with regular periodontal instrumentation has a clinical impact in terms of increased plaque accumulation and increased peri-implant tissue inflammation on zirconia implant abutments. In other words, at what point do irregularities in the abutment surface caused by peri-implant instrumentation have a direct effect on the accumulation of plaque and inflammation on the zirconium implant surface in vivo?

This study aimed to compare the effects of non-metallic hand scalers with metallic hand scalers over one year and evaluate the significance of the degree of surface alterations on the abutment/crown zirconium surface. Testing of cytokines of the peri-implant crevicular fluid was performed to determine the amplitude of inflammation. The null hypothesis is that the type and material of hand scalers will not cause an increase in surface roughness and inflammatory levels.

## 2. Materials and Methods

### 2.1. Patient Population

This randomized clinical trial was approved by the University of Manitoba’s Biomedical Research Ethics Board (HS21170 (B2017:124) and registered on Clinical-Trials.gov (NCT03316937, 11 December 2017). Patients with good oral hygiene and a long history of regular periodontal maintenance were recruited from the Dr. Sam Borden Periodontology Clinic, Dr. Gerald Niznick College of Dentistry, University of Manitoba, Winnipeg, Manitoba. Inclusion criteria were (1) good systemic health, (2) non-smokers, and (3) patients with single, screw-retained, zirconia implant-supported crowns. Patients with systemic health conditions that may affect the healing process or the outcomes of the study, patients with a history of peri-implantitis, smokers, patients with allergy or sensitivity to the materials used in the study, pregnant or breastfeeding patients, patients with insufficient oral hygiene habits, patients with other dental implants or restorations in close proximity to the study implants, and patients unable to comply with the study protocol and follow-up visits were excluded from the study. All patients were recruited by the dental receptionist or the dental hygienist and signed an informed consent form.

### 2.2. Clinical Procedure

All zirconia crowns were produced in the same lab and were glazed and polished by the same operator. A split design was selected for the study protocol. The buccal and lingual surfaces of each implant crown were randomly assigned, with simple randomization, to one of two groups. The dental hygienist, using a random number table, assigned each surface to receive scaling with either a nonmetallic scaler (Hu-Friedy Implacare IL LG1/2 non-metallic scaler) or a metallic scaler (Hu-Friedy Langer 1/2 metallic curette) after implant crown delivery. The assignment of “buccal or lingual metallic scaler” was kept sealed in the patient’s chart, so only the dental hygienist knew which side of the patient’s crown received each treatment until the crowns were reinserted at 12 months. Each patient acted as their own control and received their own scaler.

Patients received scaling and root planing at 3, 6, 9, and 12 months by an experienced calibrated dental hygienist using standardized procedures. All surfaces of the implant were debrided for 1 min using a transversal movement. Patients were instructed to use a Modified Stillman brushing technique twice per day, and cross-shoe shine flossing motion once per day. To increase compliance, each patient received oral hygiene instructions from the hygienist at the end of each maintenance therapy appointment and was provided with a three-month home care kit with dental aids, which consisted of toothpaste, a toothbrush, and implant floss.

Periodontal parameters were obtained at 0, 3, 6, 9, and 12 months by a blinded and calibrated periodontal resident. Patients were seen within one week of crown placement for baseline measurements. The parameters assessed at the implant site and patient level were modified plaque index (IPI) by Mombelli, modified gingival index (IBOP) by Mombelli, implant probing depths (PD) at six sites, presence of keratinized gingiva (KT), recession (REC), full mouth plaque index (FPI), and full mouth bleeding on probing (FBOP).

The Peri-implant Crevicular Fluid (PICF) was collected at 0, 3, and 12 months by isolating the implant site from saliva and introducing Periopaper strips into the buccal, mesial, distal and lingual sites of the implant sulcus for 30 s. The strips were placed in sealed Eppendorf tubes and transported by portable freezer to the laboratory, where they were stored at −86 degrees Celsius. The Periopaper samples were treated for the detection and quantification of the following cytokines: Interleukin-2, Interleukin-4, Interleukin-6, Interleukin-8, Interleukin-10, Tumor Necrosis Factor-alpha, and Interferon-gamma.

An MDS V-PLEX 7-plex custom panel Human Inflammatory Cytokines Kit (Rockville, MD, USA) was used in conjunction with an MSD MULTI-SPOT 96-well 10-Spot plate for the detection and quantification of the following cytokines: Interleukin-2, Interleukin-4, Interleukin-6, Interleukin-8, Interleukin-10, Tumor Necrosis Factor-alpha, and Interferon-gamma.

Periopaper samples were treated to extract the cytokines by incubating the Periopaper in 70 µL of extraction solution for one hour on ice, followed by brief centrifugation. An amount of 50 µL of the supernatant was added directly to the plate. A solution of PBS, 0.1% BSA, and 0.05% Tween-20 was used. Data were read using an MSD SECTOR Imager 2400. Units were expressed in pg/mL. Samples that did not have any detection were written as not a number (NaN) or an undefined value.

Periapical radiographs were obtained at baseline and 12 months. All radiographs were standardized by using the long cone paralleling technique utilizing XCP-ORA^®^ and XCP^®^ Positioning Systems. The distance between the implant platform and the crest of the bone was measured mesially and distally of the implant to assess crestal bone loss. After 12 months, the implant crown was removed, and a healing abutment was placed. The crown’s surface alterations were evaluated using atomic force microscopy (AFM) using the average roughness (Ra) scores. The implant crown surface was then re-polished and re-inserted. Crowns were evaluated before delivery to determine an adequate level of smoothness.

### 2.3. Atomic Force Microscope

The implant crowns were delicately taken off and examined using AFM to determine the average roughness (Ra) after a 12-month duration. Three-dimensional images were produced using AFM by scanning a sharp tip over the sample surface. A Dimension 3100 Scanning Probe Microscope (SPM) was used. Measurements were performed by the primary investigator and an engineer. NanoScope v6.13 Software was used to calculate the Ra scores. The AFM scans were performed using tapping mode, utilizing a range of cantilevers with resonant frequencies between 295 and 315 kHz. The tapping cantilevers were doped bare silicon with a tip radius of 10 nm. These cantilevers were doped but not coated. The scan rate employed was 1 Hz, determining the scan speed based on the size of the scan or image. The specific scanner used had maximum gains of 0.4 *v*/*v* for integral gain and 0.7 *v*/*v* for proportional gain. Sample sizes of 10 μm and 40 μm were randomly taken for the mesial (M), distal (D), buccal (B), and lingual (L) sides of the crown, no more the 2 mm from the crown margin. The analysis for roughness scores (Ra) only utilized the 40 μm samples. To calculate the mean roughness score (Ra), a sample size calculation was performed, determining that checking seven different spots on each side of the sample was necessary. This calculation assumed a standard deviation of 0.05 μm.

### 2.4. Statistical Methods

In total, 13 patients were recruited in this split-design study to compensate for potential dropouts. The primary outcome was the amount of surface roughness created between the metallic and non-metallic scalers. Secondary outcomes were the amplitude of inflammation based on the cytokines in the sulcus surrounding the implant, changes in probing depths, plaque score, and amount of bleeding on probing.

For comparing the longitudinal measures, mixed-effects regression models were used. Bleeding on probing is associated with a patient’s level of inflammation and, subsequently, cytokine levels, and therefore was used as a time-varying covariate to increase the statistical power. Each regression model included the predictors of treatment, time, and their reaction. The roughness measures, after the Normality test, did not follow the normal distribution. Two non-parametric tests, specifically the Kruskal–Wallis test, were conducted to analyze the data. In the initial statistical analyses, the variables under scrutiny were the type of scaler utilized (metallic, non-metallic, or both), while in the subsequent statistical analyses, the focus shifted to the surface scale (M, D, B, or L). Furthermore, post hoc tests were conducted with a significance level of α = 0.05. PROC MIXED of SAS version 9.3 was used for the analysis ††. SAS Institute Inc., Cary, NC, USA.

## 3. Results

### 3.1. Patient Characteristics

Thirteen patients, 10 females and 3 males, in the age range of 21–78 years, who had single unit screw-retained implant crowns placed by the University of Manitoba Undergraduate clinic, were included. Data from 12 patients, 3 males and 9 females, were available at the end of the study for analysis. One patient failed to report for her 6-month maintenance appointment for unknown reasons and was exited from the study. No patients reported smoking or changes in medical history throughout the study. Patients were recruited from 1 December 2017 to 5 May 2018 and were followed up until 20 June 2019, when the crowns were removed at the end of the 12 months. Each patient received a metallic and non-metallic scaler on the buccal or lingual side of their crown. Baseline demographics are the same for each patient, as each patient acted as their control.

### 3.2. Outcomes

**Figure 1:**
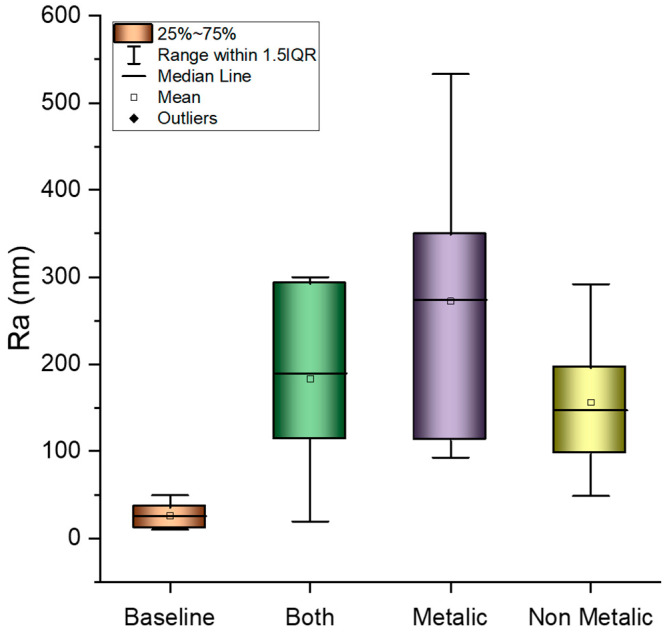
Roughness (Ra) results from the zirconia crowns after one year, according to the kind of scaler used: metallic, non-metallic, and both kinds. The baseline represents the original roughness of the crowns before treatment. The results from the Kruskal–Wallis test indicate a statistically significant (*p* < 0.05) difference between the metallic and non-metallic experimental conditions.

**Figure 2:**
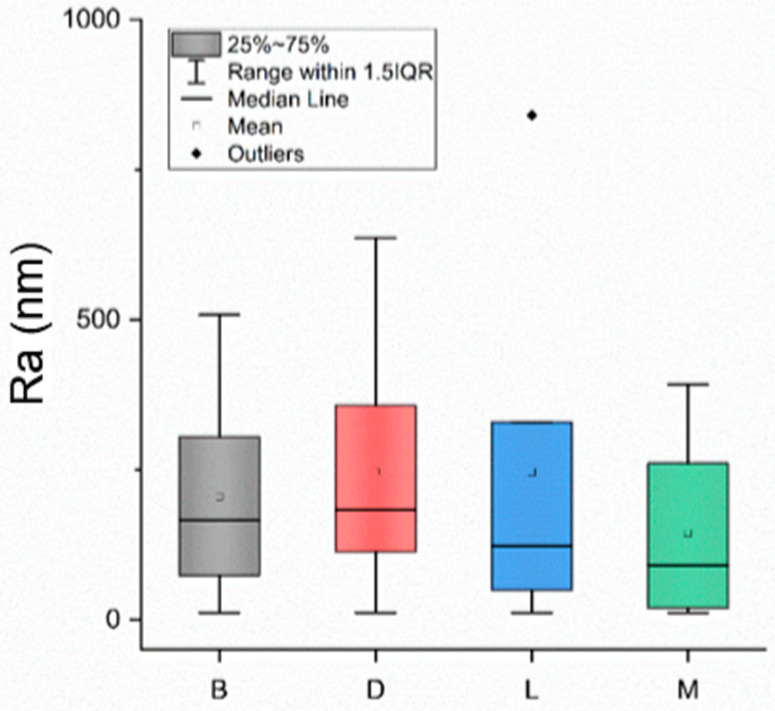
Ra results from the zirconia crowns after one year, according to the crown region: Buccal (B), Distal (D), Lingual (L), Mesial (M).

**Figure 3:**
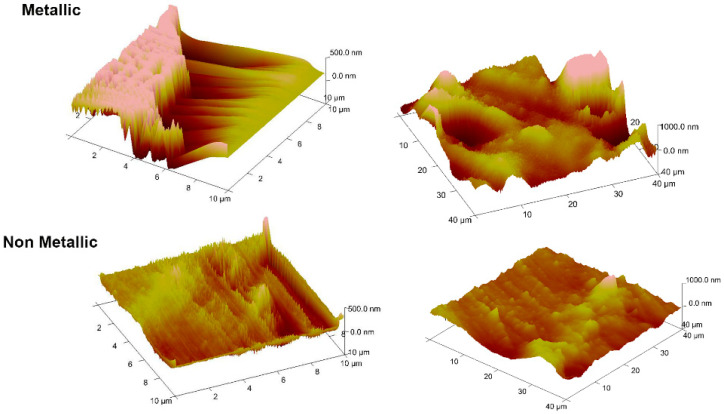
AFM images from the same sample (zirconia crown), the metallic scaler was used in the mesial and non-metallic in the lingual side (scan sizes 10 and 40 μm). The image shows deeper, broader grooves for those treated by a metallic scaler.

Figure 1 shows that there was a significant difference in surface roughness (Ra) between the metallic and non-metallic scalers, but the variable Surface was not significant statistically (Figure 2). AFM images showed relatively smooth surfaces for those treated by the non-metallic IMPLACARE^TM^ II LG1/2 scaler and rougher surfaces, with deeper, broader grooves for those treated by the metallic Langer 1/2 curette (Figure 3). The non-metallic scaler produced a median Ra value of 147.1 nm, and the metallic scaler produced a median Ra of 274 nm; when both scalers were used, the Ra mean was 189.5 nm (Figure 1). This was consistent with previous in vitro studies on titanium discs [11].

**Figure 4:**
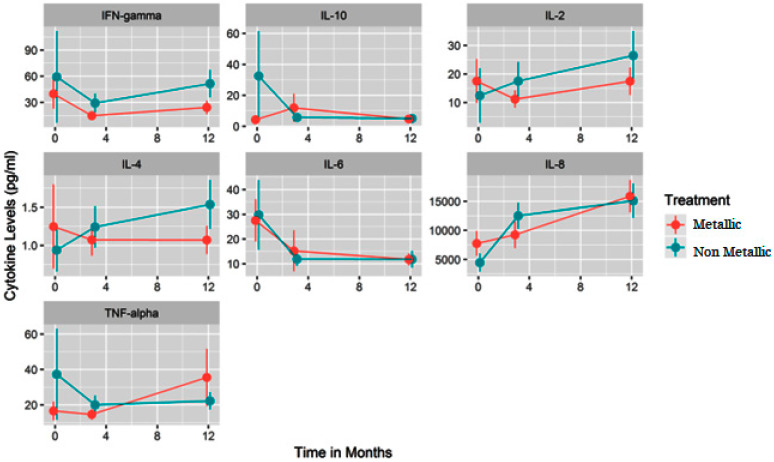
Summary of results of cytokine response of non-metallic vs. metallic scalers.

There were no significant differences between the non-metallic scaler and metallic scaler and cytokine response (Figure 4). Even though the metallic scaler produced more extensive surface alterations, there were no noted differences in the amplitude of inflammation. There was no significant difference between the two scalers and the amount of IL-2, a cytokine that promotes T-cell maturation. However, there was a significant increase in the levels of IL-2 between 0 and 12 months (*p* = 0.0135). There was no significant difference between the two scalers and the amount of IL-6, a cytokine involved in periodontal destruction, produced, but there was a significant decrease in the amount of IL-6 between 0 and 12 months, which had a drastic decrease at 3 months (*p* = 0.0376). There was no significant difference between the non-metallic and metallic scaler and the amount of IL-8, a cytokine involved in neutrophil chemotaxis and phagocytosis, produced, but there was a significant increase in the amount of IL-8 between 0 and 12 months (*p* ≤ 0.001). The non-metallic and metallic scalers did not produce a change in the amount of IL-4, IL-10, TNF-α, or IFNγ.

Finally, an attempt was made to keep the patients’ plaque accumulation and susceptibility to plaque accumulation to a minimum. The patients’ mean plaque score was 38% (SD = 15%) at baseline and 43% (SD = 14%) at 12 months. Bleeding on probing averaged under 20% for the 12 months, starting at 16% (SD = 6%) at baseline and 12% (SD = 6%) at 12 months. Patients were recalled every three months for maintenance, and oral hygiene instructions were revisited. There was an average of 4.50 mm (SD = 1.73 mm) (Table 1) of keratinized gingiva on the buccal surfaces of all the crowns. No patients reported any discomfort or difficulty in brushing or maintaining the dental implants. Periapical radiographs were obtained at baseline and 12 months. Crestal bone loss within the year was within normal limits and did not exceed 0.2 mm.

## 4. Discussion

The present blinded split-design randomized clinical trial aimed to compare the in vivo effects of non-metallic and metallic hand scalers on zirconia implant-supported crowns during a year of peri-implant maintenance. The study was designed to evaluate the degree of surface alterations on the abutment/crown zirconium surface and the cytokine levels in the peri-implant crevicular fluid to determine the extent of inflammation.

In this randomized clinical trial, clinical outcomes from the use of two different hand scalers on implant abutment surfaces were compared. Ra results were statistically different, but the level of inflammatory markers was not, so the null hypothesis was partially rejected. The non-metallic scaler Implacare IL LG1/2 scaler was selected for the study because it is made of plasteel, an unfilled resin, causing minimal alterations in vitro. The plasteel material is more rigid and less flexible than other plastic implant scalers. The metallic hand scaler, the Langer 1/2 curette, was chosen due to the fact that it is a universal curette. This metallic scaler, a stainless steel alloy, aggressively altered the surface of the metal abutment and created deeper, broader scratches, as shown by the Ra scores. The non-metallic scaler also produced an increase in surface alterations; however, it was not as aggressive. Fakhravar et al. compared metal instruments to plastic instruments, in vitro, on titanium abutments. They also confirmed that plastic scalers induced roughness on the abutment surfaces, but the metal scalers induced more surface damage. They credited this difference to the sharp cutting metal blade of the metal scaler. The authors stated that the surface changes in the plastic scaler, a universal scaler from Hu Friedy, could be explained by plastic particles and debris that were left behind on the titanium abutment surface [14]. However, in our study, there were no plastic particles left behind on the zirconium abutments after use with the non-metallic scaler.

Titanium has been widely used for implant abutments due to its strength, resistance to malformation, and the opportunity to fabricate as one piece. However, titanium abutments can produce a grayish appearance in the overlying tissue. Zirconia abutments offer a more esthetic alternative, especially in esthetic cases or in patients with a thin biotype. Moreover, in vivo, zirconia appears to be less prone to bacterial adhesion compared to titanium. As zirconia is made of ceramic and will not corrode, it could account for healthier gingival tissues around an implant.

The instruments used for the debridement of dental implants should be efficient and effective in removing plaque and calculus while minimizing damage to the implant surface. The present study demonstrated that non-metallic hand scalers caused less surface alteration on zirconia implant-supported crowns compared to metallic hand scalers during a year of peri-implant maintenance. The results suggest that non-metallic hand scalers may be a better option for the debridement of zirconia implant-supported crowns due to their ability to remove plaque and calculus while causing minimal surface damage.

Prior studies have investigated surface alterations on titanium and titanium discs. These in vitro studies demonstrated that the metallic hand scaler produced surface alterations, which coincided with our study [11,12,13,14,15]. It is well known that stainless steel scalers produce more alterations to the titanium implant abutment surface, and by extension, it could be extrapolated that this could make an implant surface more susceptible to plaque and calculus accumulation and therefore induce more inflammation. Hallmon et al. compared metallic, non-metallic, and sonic instrumentation on titanium abutments and also concluded that the metallic scalers produced deeper grooves, scratches, and surface alterations [20]. They also concluded that surface roughening could be consistent with plaque growth rates. However, our patients were on a 3-month periodontal maintenance, and the amount of surface irregularities was not coincident with plaque growth.

Moreover, the cytokine levels in the peri-implant crevicular fluid were lower in patients treated with non-metallic hand scalers, indicating less inflammation. This finding is consistent with previous studies that have shown that metallic instruments can cause more inflammation due to their ability to generate more heat and vibration during use.

The difference between past studies and the current one is that they were not in vivo and did not demonstrate whether surface alterations affected inflammatory indices. In this study, however, there were no significant differences that were found in the levels of cytokines IL-2, IL-4, IL-6, IL-8, IL-10, TNF-α, or INFγ between the non-metallic and metallic scalers. This demonstrated that under routine 3-month maintenance, the abutment surface roughness caused by metallic scalers did not produce a statistically significant increase in inflammation in the implant sulcus. Even though the metallic scaler produced deeper, broader scratches on the abutment surface in a patient under regular maintenance, this did not produce an increase in inflammatory cytokines. IL-6, associated with periodontal destruction and bone resorption, actually significantly decreased in the subjects from 0 to 12 months, which was seen immediately after delivery of oral hygiene instructions and dental aids between baseline and 3 months. Systematic reviews have researched the role of cytokines in distinguishing peri-implant disease and have determined that further research is needed [26,27,28,29]. Ghassib et al. studied IL-8 and IL-6 and concluded that the pro-inflammatory cytokines can be used as an adjunct tool but that there was only moderate evidence to support this [26]. A systematic review completed by Duarte et al. established that proinflammatory cytokines, such as IL-8, demonstrated increased levels in implants with peri-implantitis compared to healthy implants. They also determined that proinflammatory cytokines are the most promising proteins to use for biomarkers but that there was only moderate evidence to support this, and further investigation was needed [27]. The concept of gingival crevicular fluid (GCF) having different compositions on either side of a dental implant may seem complex, but recent research indicates that it is indeed the case [29,30,31]. Factors such as inflammation, implant loading, oral hygiene, and individual variations can all contribute to differences in GCF composition. For instance, if there is inflammation or infection on one side of the implant, the GCF will contain higher levels of inflammatory markers. Similarly, if the implant is unequally loaded or if there are differences in oral hygiene practices, it can affect the GCF composition. Moreover, individual variations in how each person’s body reacts to implants can also play a role in the differences observed in GCF composition. These findings highlight the importance of monitoring GCF levels on both sides of a dental implant to ensure optimal oral health [32,33,34,35,36].

Previous in vitro studies showed that metallic scalers debride the abutment surface more effectively and efficiently [16,17]. Patients were instructed to use the oral hygiene aids given and had their plaque and bleeding on probing scores monitored. The mean bleeding on probing score ranged from 16% at 0 months to 12% at 12 months, and the mean plaque score was 43% (SD = 14%) at 12 months. Patients were also seen under 3-month maintenance appointments, and levels of inflammation were kept at a minimum around the implant abutment. Using non-metallic scalers preserved the implant abutment surface, and the implant sulcus remained healthy. However, due to the patient’s regular maintenance, the scratched and damaged abutment surface had a minimal inflammatory effect on the implant sulcus.

Patients were treated by one hygienist, who administered the same number of strokes per side under the same pressure, and measurements were taken by a standardized periodontal resident to attempt to eliminate bias. Even though efforts were made to increase compliance with the patients, there could have been variations in oral hygiene practices that could affect the results. Other potential limitations of this study could be the small sample size to detect differences in peri-implant inflammation and only two scalers compared. Different scaler tips are made of different manufacturing materials, have different shapes and designs, have different degrees of flexibility, and have variations in contact angle. However, scalers were chosen that are used frequently today in North America. Patients were seen every 3 months for maintenance in a university setting; however, this may not be realistic in private practice. Patients are often seen every 6 months, which may affect the level of inflammation. Also, in this study, patients were followed over a 12-month period. The longer-term effects on the implant abutment could be more detrimental.

While this study provides valuable insights into the use of non-metallic hand scalers for the debridement of zirconia implant-supported crowns, further research is needed to determine the long-term effects of non-metallic hand scalers on peri-implant tissue health. Additionally, it would be interesting to investigate the use of other non-metallic instruments, such as ultrasonic scalers, for the debridement of dental implants. The use of non-metallic hand scalers during peri-implant maintenance may be beneficial for patients with zirconia implant-supported crowns due to their ability to remove plaque and calculus while causing minimal surface damage and inflammation. Further research is needed to determine the long-term effects of non-metallic instruments on peri-implant tissue health.

Despite the increasing use of Zirconia crowns in implant dentistry, there is very limited information available on how to properly maintain it. This lack of information could potentially lead to complications and failures in the long-term use of Zirconia crowns [32]. It is important for dental professionals to be aware of this limitation and to continue researching and studying the proper maintenance techniques for this material. As technology and materials continue to advance, it is crucial that dental professionals stay informed and educated to provide the best possible care for their patients.

## 5. Conclusions

From this randomized clinical trial, it can be concluded that stainless steel metallic scalers produce deeper, more aggressive surface alterations to the abutment/crown zirconia surface in vivo in zirconia abutments. However, there was no statistically significant difference between the degree of surface alterations and the amplitude of cytokine inflammation produced while patients were on regular maintenance therapy for over a year.

## Figures and Tables

**Table 1:** Average values of patients’ oral hygiene conditions.

Time	Full Mouth Mean Plaque Score	Full Mouth Mean BOP	Mean Keratinized Gingiva around Implant
0 months	35% ± 15%	16% ± 6%	4.50 mm ± 1.73 mm
3 months	49% ± 19%	16% ± 6%	4.00 mm ± 1.62 mm
6 months	52% ± 18%	17% ± 8%	4.75 mm ± 1.71 mm
9 months	44% ± 18%	15% ± 9%	4.67 mm ± 1.50 mm
12 months	43% ± 14%	12% ± 6%	4.67 mm ± 1.61 mm

## Data Availability

Data supporting reported results could be sent upon request.
